# The Dual Roles of Gut Microbiota in Biliary Atresia: Mechanisms, Biomarker Potential, and Therapeutic Implications

**DOI:** 10.3390/microorganisms14051076

**Published:** 2026-05-09

**Authors:** Jianing Yan, Li Jiang, Yating Li, Hui Lv, Wenrui Wu, Liya Yang, Jianing Chen, Ding Shi

**Affiliations:** 1 State Key Laboratory for Diagnosis and Treatment of Infectious Diseases, National Clinical Research Center for Infectious Diseases, Collaborative Innovation Center for Diagnosis and Treatment of Infectious Diseases, The First Affiliated Hospital, College of Medicine, Zhejiang University, Hangzhou 310000, China; 2 Health Promotion Center, Zhejiang Provincial People’s Hospital, People’s Hospital of Hangzhou Medical College, Hangzhou 310000, China

**Keywords:** biliary atresia, gastrointestinal microbiome, gut–liver axis, biomarkers, dysbiosis, therapeutics

## Abstract

Biliary atresia (BA) is a progressive fibroinflammatory cholangiopathy of infancy that rapidly advances to cholestasis, fibrosis, cirrhosis, and liver failure if bile drainage is not restored early. Although Kasai hepatoportoenterostomy (KPE) remains the standard first–line operation, many children still develop recurrent cholangitis, persistent cholestasis, and progressive native liver injury. Increasing evidence indicates that the gut microbiota participates in this clinical course through the gut–liver axis. In BA, dysbiosis may weaken the intestinal barrier, increase translocation of microbe–associated molecular patterns (MAMPs), amplify innate and adaptive immune activation, disturb bile acid signaling, and promote fibrogenic and ferroptosis–related injury. In contrast, beneficial taxa and their metabolites may preserve epithelial integrity, support immune tolerance, maintain bile acid homeostasis, and constrain oxidative stress. This review summarizes current evidence on these contrasting harmful and protective effects, stage–specific microbiome signatures reported before and after KPE, and critically evaluates the present status of microbiota–based biomarkers and interventions. We emphasize that although several microbial signatures and therapeutic approaches are promising, they are not yet ready for routine clinical implementation and require prospective validation with standardized endpoints.

## 1. Introduction

**Figure 1:**
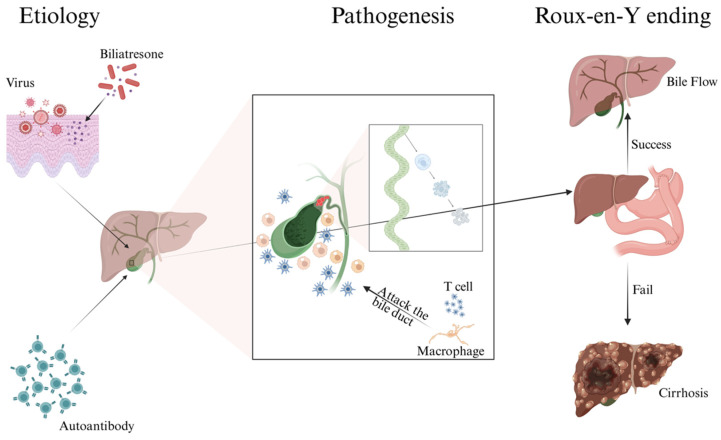
Clinical context of BA. Early bile duct injury leads to cholestasis and progressive fibrosis; after KPE, children may follow divergent trajectories toward jaundice clearance and native liver survival or toward recurrent cholangitis, cirrhosis, and liver transplantation.

Biliary atresia (BA) is a rare but devastating neonatal disorder characterized by progressive fibro–obliterative injury of the extrahepatic and intrahepatic bile ducts [1,2,3]. Even when the Kasai procedure is performed in a timely manner, postoperative outcomes vary widely, and many infants continue to develop recurrent cholangitis, portal fibrosis, cirrhosis, and eventual need for liver transplantation [4,5,6]. This gap between short–term bile drainage and durable native liver survival has intensified interest in adjunctive mechanisms that shape disease progression after surgery (Figure 1).

One such mechanism is the gut–liver axis. The intestinal microbiota regulates barrier integrity, immune education, bile acid biotransformation, and microbial metabolite production, all of which are highly relevant in cholestatic liver disease [7,8,9]. Pediatric BA cohorts consistently report dysbiosis marked by expansion of pathobionts such as Streptococcus, Klebsiella, Enterococcus, and Veillonella, together with depletion of taxa commonly linked to butyrate production, bile acid conversion, and epithelial homeostasis [10,11]. These shifts correlate with liver biochemistry, fibrosis markers, bile acid profiles, jaundice clearance, and postoperative cholangitis.

The literature points to two opposite patterns of microbiota–host interaction. One pattern is associated with barrier leak, portal delivery of MAMPs, inflammatory amplification, bile acid disequilibrium, and fibrosis. The other is associated with preservation of the mucus layer and tight junctions, regulatory immune responses, and reduced oxidative and fibrotic injury. This arrangement reduces overlap between mechanistic themes and better reflects how the microbiota may influence BA progression across disease stages.

In this review, we first summarize clinical microbiome studies before and after KPE, and then discuss the harmful and protective roles of the gut microbiota in matched mechanistic domains: barrier integrity, immune regulation, bile acid biology, fibrosis, and ferroptosis–related injury. We then evaluate microbiota–derived biomarkers and microbiota–targeted interventions, with particular attention to study size, endpoints, timing, and the current lack of validated hard clinical outcomes. This structure is intended to provide a more direct mechanistic interpretation of the available evidence.

## 2. The Gut–Liver Axis: A Vital Connection

The gut–liver axis is a bidirectional system linking the intestine and liver through portal blood flow, immune signaling, and enterohepatic bile acid circulation [12,13,14,15,16]. Under physiological conditions, this network limits pathogen expansion, supports epithelial integrity, and maintains a bile acid pool that is continuously modified by gut microbes.

In BA, persistent cholestasis disrupts this equilibrium. Reduced bile delivery to the intestine weakens the antimicrobial and signaling functions of bile acids, favors dysbiosis, and impairs intestinal barrier maintenance [17,18,19]. At the same time, altered microbial composition feeds back on bile acid transformation and immune tone, increasing the portal exposure of the liver to inflammatory microbial products and toxic bile acid species.

In BA, the gut–liver axis is not a generic background concept but a mechanistic bridge connecting cholestasis to microbial reshaping, barrier dysfunction, immune dysregulation, and fibrosis [20,21,22,23,24]. The following sections use this axis to interpret both injurious and protective microbiota–related processes.

## 3. Alterations of Gut Microbiota in Biliary Atresia: Evidence from Clinical Studies

Clinical studies summarized in Table 1 show that BA is associated with reproducible, although not fully uniform, microbiome alterations. Before KPE, most cohorts report reduced diversity or clear community separation from controls, with expansion of Streptococcus, Klebsiella, Enterococcus, Escherichia–Shigella, or Veillonella, and depletion of Bacteroides, Faecalibacterium, Bifidobacterium, or other commensals linked to short–chain fatty acid (SCFA) generation and barrier support [11,25,26,27,28,29,30].

After KPE, the microbiota remains dynamic rather than normalized. Some postoperative profiles shift toward higher abundance of Bifidobacterium, Blautia, Lachnospiraceae, or Akkermansia and correlate with better bile flow, lower bilirubin, lower gamma–glutamyl transferase (GGT), or lower fibrosis indices [26,31,32,33,34]. However, postoperative dysbiosis can persist or recur, and cholangitis–prone or jaundice–nonclearance groups continue to show enrichment of potentially harmful taxa [26,27,35].

Interpretation remains limited by heterogeneous study design, small sample sizes, mixed pre– and post–KPE populations, and inconsistent sequencing pipelines. Nevertheless, the overall pattern is coherent: BA is characterized by a stage–dependent loss of microbial functions associated with barrier integrity, bile acid conversion, and immune homeostasis, together with an increase in taxa compatible with inflammatory and cholangitic risk.

**Table 1:** Key clinical microbiome studies in BA, organized by stage relative to KPE.

Stage	Participants	Platform/Sample	Main Harmful–Associated Findings	Main Protective–Associated Findings	Clinical Note	Ref.
Pre–KPE	BA(11), CD(6), HC(6)	16S; feces	Reduced diversity; increased Proteobacteria, Streptococcus, and Lactobacillus	Relative preservation of selected commensals varied by comparator group	Low serum butyrate; diagnostic ratios showed promise but were derived from a small cohort	[18]
Pre–KPE	BA(34), HC(34)	16S + metagenomics; feces	Lower diversity; enrichment of Streptococcus, Klebsiella, Enterococcus, and Lactobacillus; reduced bile acids	Lower Bifidobacterium and other butyrate–producing taxa in BA	Streptococcus/Bacteroides ratio showed diagnostic potential; no external validation	[30]
Pre–KPE	Early BA(16), HC(16); late BA(16), HC(10)	Metagenomics; feces	Persistent Proteobacteria–, Klebsiella–, Streptococcus–, and Veillonella–rich signatures; enriched pathogen–associated functions	Relative enrichment of Bifidobacterium/Blautia in early–stage and Bacteroidetes/Verrucomicrobia in late–stage subgroups	Secondary bile acid perturbation and functional signals of pathogen invasion	[25]
Pre–KPE	BA(46), HC(38)	16S; feces	Escherichia–Shigella, Streptococcus, Veillonella, and Citrobacter expansion	Bifidobacterium, Faecalibacterium, Blautia, and Agathobacter relatively enriched in comparator groups	Links between dysbiosis, liver enzymes, bilirubin, and steroid/bile–acid changes	[26]
Pre–KPE	BA(17), CD(8); BA(31), HC(20); bile–flow subgroup study	16S/WGS; feces	Shigella, Klebsiella, Veillonella, and Firmicutes/Fusobacteria enrichment in unfavorable profiles	Bifidobacterium enrichment associated with very good bile flow or less severe injury	Suggests microbial patterns differ by bile–flow status and disease severity	[28,33,36]
Post–KPE	BA(42), HC(40)	Metagenomics; feces	Streptococcus, Klebsiella, Enterococcus, and Veillonella remained enriched in unfavorable profiles	Bifidobacterium, Blautia, Lachnospiraceae, and B. longum associated with better postoperative recovery	Higher B. longum correlated with lower bilirubin, AST, and GGT	[34]
Post–KPE	BA(55), HC(19), CD(21)	16S; feces	Enterococcus, Clostridium, Fusobacterium, and Pseudomonas enrichment	Bifidobacterium enrichment	Low acetate and altered propionate/butyrate profile; microbiota richness inversely related to cholestasis/fibrosis severity	[31]
Post–KPE	Longitudinal and subgroup cohorts after KPE	16S/metagenomics; feces	Persistent pathobiont enrichment in cholangitis, recurrent cholangitis, or jaundice–nonclearance subgroups	Bifidobacterium–dominant profiles associated with better liver biochemistry and bile flow	Postoperative microbiota remains dynamic rather than normalized	[26,27,32,33,35]

## 4. Pathogenic Effects of Gut Microbiota Dysregulation in BA

When dysbiosis predominates, the microbiota may amplify BA progression through three interlocking domains: barrier failure and microbial translocation; proinflammatory immune skewing; and profibrotic or bile acid–related injury.

### 4.1. Barrier Dysfunction and Microbial Translocation

A major mechanism in this setting is loss of intestinal barrier integrity. Under healthy conditions, commensals from the Firmicutes and Bacteroidetes phyla support mucus production, epithelial renewal, and tight–junction stability [37]. In BA and other cholestatic states, dysbiosis and reduced luminal bile acids weaken this barrier and facilitate translocation of MAMPs such as lipopolysaccharide (LPS) into portal blood [38,39,40]. The resulting cycle of epithelial injury, inflammatory signaling, and further dysbiosis directly links the diseased intestine to ongoing liver injury [41].

### 4.2. Proinflammatory and Dysregulated Immune Responses

**Figure 2:**
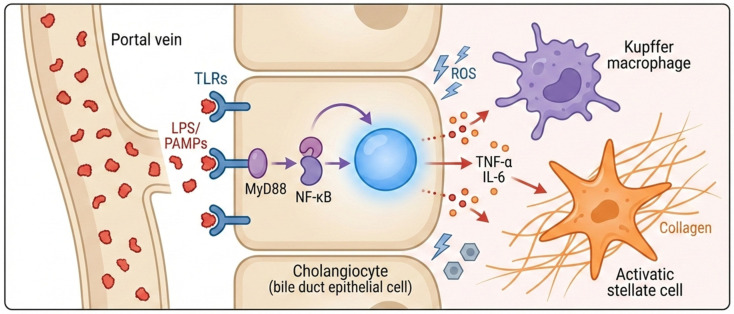
Inflammatory cascade triggered by barrier injury and microbial translocation. Barrier injury increases portal delivery of MAMPs, activates hepatic pattern–recognition pathways, and drives cytokine release, cholangiocyte injury, stellate–cell activation, and progressive fibrosis.

Once microbial products reach the liver, they engage pattern–recognition receptors, including toll–like receptors (TLRs) and NOD–like receptors (NLRs) on immune and parenchymal cells. This activates MyD88–NF–kB signaling and promotes cytokines such as tumor necrosis factor–alpha (TNF–alpha), interleukin (IL)–1beta, IL–6, IL–8, and IL–15, thereby recruiting dendritic cells, macrophages, and natural killer (NK) cells to the portal and biliary compartment [42,43,44,45,46,47,48]. In parallel, inflammasome activity and barrier–protein loss prolong exposure to gut–derived signals and sustain cholangiocyte injury [49,50,51] (Figure 2).

Adaptive immunity is also reshaped in a pathogenic direction. BA tissues show increased Th1–associated signaling, especially interferon–gamma, and impaired regulatory T–cell control [4,52,53,54,55,56,57,58,59,60,61]. This combination favors persistent cytotoxic inflammation rather than resolution. Humoral abnormalities may contribute as well, because altered IgG and IgM profiles have been linked to prognosis, although the mechanistic role of B–cell responses remain less well defined [62].

This immune phenotype should not be described as exclusively Th1–driven. Th2–associated cytokines such as IL–4 and IL–13 is also increased in some models and may interact with profibrotic pathways [56,63]. Dysbiosis in BA is better understood as causing immune dysregulation, with excessive inflammatory activation and insufficient regulatory counterbalance.

The gut–liver interface can shift from a tolerance–supporting system under physiological conditions to a route of persistent inflammatory amplification when barrier failure and dysbiosis dominate.

### 4.3. Profibrotic, Bile Acid–Related, and Ferroptosis–Linked Injury

The downstream consequence of persistent immune activation is biliary fibrosis. NK–cell–mediated epithelial injury, ongoing cholangiocyte stress, and stellate–cell activation converges on transforming growth factor–beta (TGF–beta)–centered fibrogenesis, promoting extracellular matrix deposition and progressive architectural distortion [63,64,65,66,67,68,69]. Loss of antifibrotic immune restraint may further worsen this process.

Bile acid dysregulation is tightly interwoven with this profibrotic state. In BA cohorts, specific taxa correlate with altered primary–to–secondary bile acid ratios and with serum total bile acid burden [26,30,70]. These changes can impair farnesoid X receptor (FXR) and Takeda G protein–coupled receptor 5 (TGR5) signaling, weaken colonization resistance, and sustain both epithelial stress and inflammatory signaling [12,71,72]. These results indicate that microbiota–associated bile acid disequilibrium is part of the pathogenic process rather than a background consequence of cholestasis alone.

Ferroptosis adds another layer of injury. Experimental evidence indicates that iron–dependent lipid peroxidation can aggravate cholangiocyte and hepatocyte damage in cholestatic settings [73]. Gut–derived inflammatory signals such as LPS may intensify reactive oxygen species generation and increase ferroptotic susceptibility [74,75]. Although direct BA–specific evidence remains limited, ferroptosis plausibly links dysbiosis, oxidative injury, and fibrosis progression.

In BA, dysbiosis contributes to a linked pathogenic sequence in which barrier dysfunction permits translocation, translocation drives immune injury, and immune–metabolic injury promotes fibrosis, bile acid disruption, and ferroptosis.

The same mechanistic themes recur across studies because they are not isolated phenomena but mutually reinforcing components of disease progression.

Therapeutic strategies should aim not only to suppress pathogens but also to restore the protective microbial functions that are lost in BA.

## 5. Protective or Homeostatic Functions of the Gut Microbiota in BA

### 5.1. Barrier Protection and Colonization Resistance

Protective microbial functions along the gut–liver axis are primarily linked to preservation of epithelial integrity and colonization resistance. SCFA–producing commensals such as Bacteroides, Bifidobacterium, and selected Clostridial taxa generate acetate, propionate, and butyrate, which nourish colonocytes, promote mucus stability, and support tight–junction maintenance [76,77,78,79,80,81,82]. In this setting, the microbiota acts as a barrier organ that restricts portal access of inflammatory microbial products.

Probiotics may reinforce this barrier effect through several complementary mechanisms, including competitive exclusion of pathobionts, stimulation of mucosal defenses, and support of local bile acid homeostasis [83,84,85,86]. These effects are stage–dependent: protection is not defined simply by the absence of pathogens, but by the presence of communities and metabolites that resist translocation and preserve epithelial resilience.

### 5.2. Regulatory Immune and Anti–Inflammatory Effects

A second protective mechanism is the regulation of immune tone. SCFAs signal through G–protein–coupled receptors and histone deacetylase inhibition to increase IL–10, restrain NF–kB–dependent inflammatory programs, and promote Foxp3–positive regulatory T–cell differentiation [87,88,89,90,91,92,93]. These pathways counter the exaggerated inflammatory responses that characterize BA and help explain why loss of butyrate–producing taxa may be clinically relevant.

Probiotic strains can add to this regulatory environment by modulating antigen presentation, secretory IgA, and mucosal immune communication [94,95,96]. Their effects are strain–specific rather than class–wide, which is why clinical interpretation should focus on the exact organism, timing, and endpoint studied rather than on the generic label ‘probiotic.’

Because immune immaturity in early infancy may make the same intervention helpful in one postoperative context but ineffective in another, these immune effects should be interpreted as conditional and context–dependent.

Even so, the general direction of the evidence is consistent: microbial communities enriched in Bifidobacterium, Blautia, or other SCFA–linked taxa are repeatedly associated with a less inflammatory postoperative phenotype.

Restoring immunoregulatory microbial functions may be as important as eliminating overt pathogens.

### 5.3. Antifibrotic, Bile Acid–Modulating, and Antioxidative Effects

**Figure 3:**
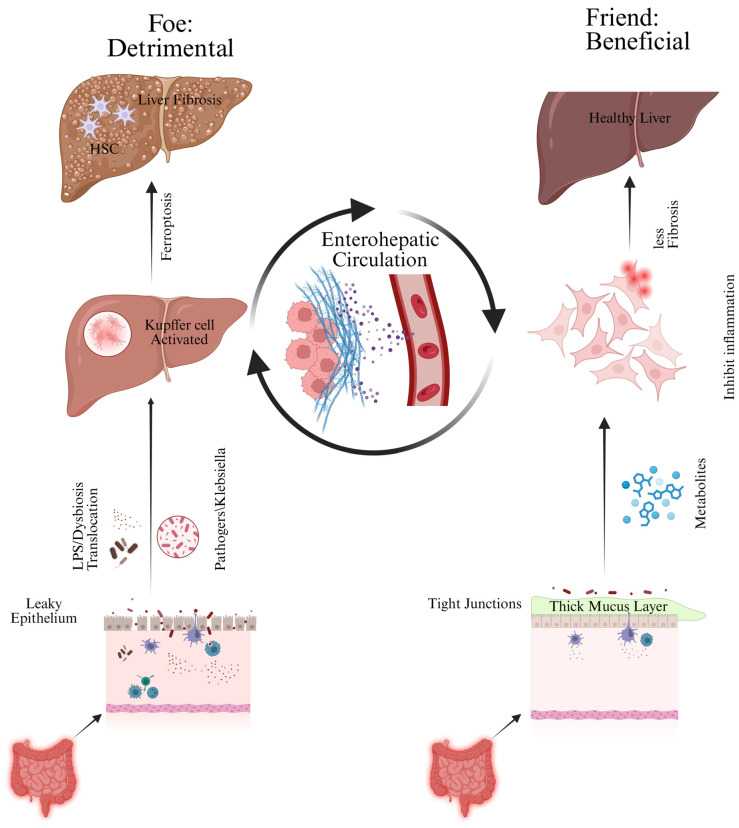
Harmful and protective microbiota–related effects in BA. Dysbiosis promotes barrier leak, MAMP translocation, inflammation, and fibrosis, whereas beneficial communities preserve epithelial integrity, immune tolerance, and metabolite–mediated protection.

**Figure 4:**
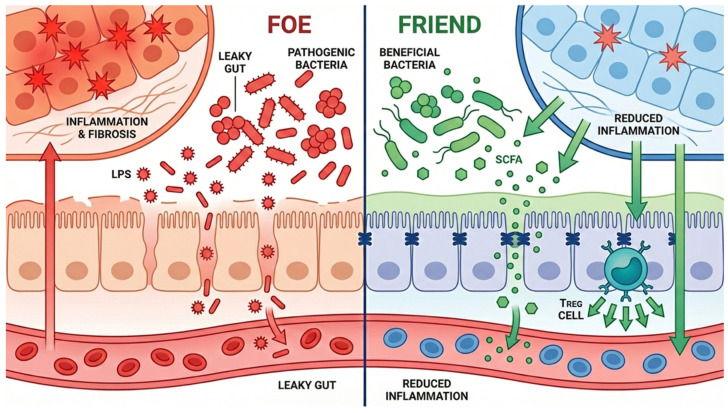
Matched harmful and protective mechanisms along the gut–liver axis. The left side emphasizes barrier failure, inflammatory amplification, and fibrosis, whereas the right side emphasizes barrier restoration, SCFA support, and regulatory immune signaling.

Protective microbiota–related effects also extend to fibrosis, bile acid signaling, and oxidative stress. Butyrate and other microbial metabolites can suppress stellate–cell activation, improve epithelial stress responses, and reduce oxidative injury in experimental systems [80,89,97]. Beneficial microbes may also promote a bile acid profile that better supports FXR/TGR5 signaling and mucosal homeostasis [70,71,72,86]. In BA, evidence for direct effects on native liver survival or transplant–free survival remains limited, and neonatal dosing, timing, and safety windows are not yet standardized (Figure 3 and Figure 4).

## 6. Gut Microbiota as Diagnostic and Prognostic Biomarkers

Several studies suggest that microbial signatures may help distinguish BA from other cholestatic disorders, but their present value is exploratory rather than practice–changing. In a combined public–data and clinical analysis, 43 BA, 33 disease–control, and 42 healthy–control samples were first examined in silico, followed by a 16S cohort of 11 BA, six disease–control, and six healthy–control samples [18]. In that study, the Streptococcus/Bacteroides ratio discriminated BA from disease controls with an area under the curve (AUC) of 0.9035, and the Streptococcus/Eggerthella ratio yielded an AUC of 0.8333 [18]. These metrics are encouraging, but they come from small datasets without external validation or implementation–ready thresholds.

Other cohorts reinforce the same message but provide less complete performance reporting. A study of 34 BA and 34 healthy controls showed that the Streptococcus/Bacteroides ratio had diagnostic promise, while postoperative studies associated Bifidobacterium–dominant profiles or higher Bifidobacterium longum abundance with better bile flow, lower bilirubin, lower GGT, and less severe liver injury [30,33,34,98]. These findings suggest prognostic value, but they remain associative and may reflect disease stage, feeding patterns, antibiotic exposure, or operative status.

Therefore, microbiome–based indices are not yet ready for clinical implementation in BA. Before they can be used for diagnosis or risk stratification, studies will need larger multicenter cohorts, prespecified cutoffs, external validation, and comparison against existing clinical workflows.

## 7. Therapeutic Implications: Targeting the Gut Microbiota

**Figure 5:**
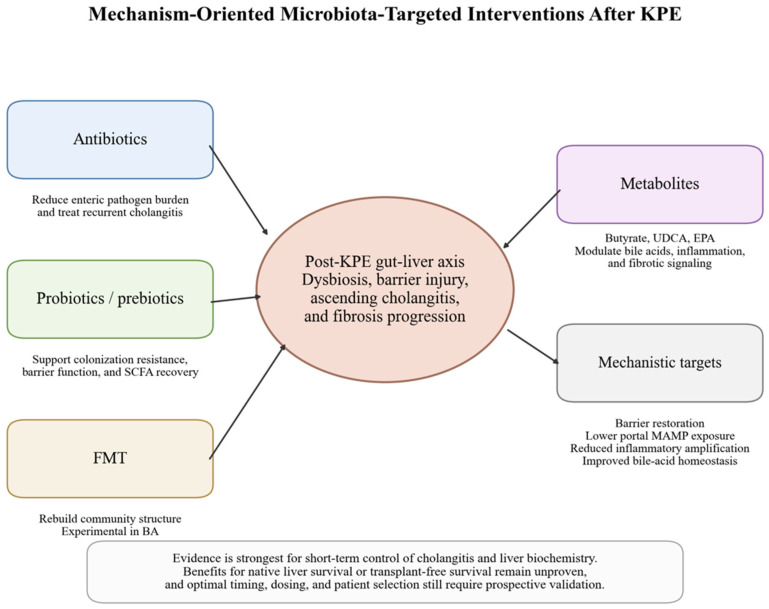
Microbiota–targeted interventions after KPE. Candidate approaches include antibiotics, probiotics, fecal microbiota transplantation, and metabolite–based strategies that aim to reduce pathogen burden, improve barrier function, restore microbial homeostasis, and attenuate inflammatory–fibrotic injury.

Microbiota–targeted therapy in BA is clinically attractive because recurrent cholangitis and persistent dysbiosis remain common after KPE. However, the therapeutic literature is heterogeneous, and the strongest evidence still concerns short–term biochemical or cholangitis–related endpoints rather than hard outcomes such as native liver survival (Figure 5 and Table 2).

### 7.1. Antibiotics

Antibiotics remain the most established microbiota–modifying therapy after KPE because they directly target ascending cholangitis. Their benefit is clearest when used to control overt infection. In a multicenter prospective trial, meropenem or cefoperazone–sulbactam was used as first–line therapy for mild cholangitis, while meropenem plus intravenous immunoglobulin was used in severe disease; the main reported endpoints were control of infection, improvement in inflammatory and liver biochemical markers, and shorter hospital stay [99]. These data support antibiotics for treatment of cholangitis, but not necessarily for prolonged microbiome–directed prophylaxis.

The limitation is a biological trade–off. Prolonged or repeated exposure may deplete beneficial taxa such as Bifidobacterium and select for antimicrobial resistance [100,101,102]. Thus, antibiotic regimens should be framed as necessary anti–infective therapy whose microbiome cost must be minimized, not as a simple route to eubiosis.

Future studies should therefore report both infection–related endpoints and microbiome consequences, ideally with native liver survival or transplantation–free survival as downstream outcomes.

### 7.2. Fecal Microbiota Transplantation (FMT)

FMT is conceptually appealing because it aims to rebuild microbial community structure rather than merely suppress pathogens. Yet in BA, evidence remains preclinical or theoretical. Clinical experience comes mainly from other conditions, such as recurrent Clostridioides difficile infection or pilot work in primary sclerosing cholangitis [103,104]. For infants with BA, donor screening, transmission risk, and immune vulnerability remain major barriers, so FMT should still be considered experimental in this setting.

### 7.3. Probiotics, Prebiotics, and Synbiotics

Probiotic studies in BA illustrate both promise and uncertainty. In a pilot study of 20 jaundice–free children aged 0–3 years after Kasai operation, neomycin prophylaxis (25 mg/kg/day for 4 days per week) was compared with a fixed daily Lactobacillus casei rhamnosus regimen for 6 months; the primary endpoint was recurrent cholangitis, and both active groups performed similarly and better than historical controls [105]. In that context, probiotic therapy appeared feasible and potentially useful for cholangitis prevention.

However, a later double–blind placebo–controlled trial randomized 14 post–hepatoportoenterostomy patients to LGG and 16 to placebo for 6 months, with bacterial cholangitis, laboratory indices, microbiota composition, and SCFAs as the main endpoints and liver transplantation assessed during 2–year follow–up [106]. LGG was associated with numerically fewer cholangitis episodes (21% vs. 50%), but the difference was not statistically significant, and neither laboratory outcomes, microbiota composition, nor transplantation rates improved significantly. These conflicting results underscore that probiotic efficacy is strain–, timing–, and endpoint–dependent.

### 7.4. Metabolite Interventions

Metabolite–based therapy may ultimately offer better dosing control than live–microbe interventions, but BA data are still limited. Butyrate is the most discussed candidate. Experimental work suggests that butyrate supplementation can reduce bile duct inflammation, support a more favorable microbial configuration, and attenuate fibrotic signaling [27,107,108]. However, these studies are preclinical; standardized pediatric dosing, optimal timing relative to KPE, and effects on hard endpoints such as native liver survival or transplantation–free survival are not available.

UDCA provides a more clinically mature but also more conflicted example. In a prospective postoperative study, 16 children who had successful portoenterostomy received UDCA for at least 18 months; discontinuation worsened ALT, AST, and GGT, and liver function improved after treatment was resumed [109]. By contrast, a historical cohort of 141 infants reported that 108 children who received UDCA at 20 mg/kg/day for a mean of 252.6 days did not achieve better outcomes, and the absence of UDCA was associated with a higher likelihood of a successful outcome [110]. These discordant data suggest that timing, stage, and patient selection matter, and that UDCA cannot be assumed to improve hard outcomes simply because it improves bile biochemistry in selected postoperative patients.

Other metabolite–directed strategies, including eicosapentaenoic acid and D–2–hydroxyglutarate–related pathways, remain intriguing but early [111,112]. At present, they should be viewed as mechanistically informative rather than clinically established.

Overall, microbiota–targeted interventions are best understood as adjunctive and stage–specific. The field now needs trials that define timing relative to KPE, dosing, duration, and hard clinical endpoints rather than only short–term laboratory change.

**Table 2:** Microbiota–targeted interventions in BA: timing, endpoints, and present limitations.

Strategy	Timing/Regimen Where Available	Primary Endpoints or Main Reported Outcomes	Interpretation
Antibiotics	Post–KPE prophylaxis or treatment; examples include ceftriaxone, cefoperazone, meropenem, and meropenem plus IVIG for severe cholangitis	Mainly cholangitis control, laboratory improvement, and shorter hospital stay [99,113,114,115]	Useful for infection control, but prolonged exposure may worsen dysbiosis and select resistance; benefit on native liver survival is unproven
LGG/probiotic prophylaxis (pilot study)	Twenty jaundice–free children aged 0–3 years after Kasai; neomycin 25 mg/kg/day for 4 days/week vs. a fixed daily Lactobacillus casei rhamnosus regimen for 6 months [105]	Recurrent cholangitis during treatment; stool culture changes; weight gain [105]	LGG appeared similar to neomycin and better than historical controls, but the study was small and used no hard survival endpoint
LGG/probiotic prophylaxis (placebo–controlled trial)	LGG(*n* = 14) vs. placebo(*n* = 16) for 6 months after HPE [106]	Bacterial cholangitis, laboratory markers, microbiota composition, SCFAs, and 2–year transplantation follow–up [106]	Numerically fewer cholangitis episodes with LGG, but no statistically significant improvement in labs, microbiota, or transplantation rate
FMT	Investigational; no established pediatric BA regimen	Rationale based on microbiota restoration; no mature BA outcome data yet [104]	Not ready for routine use in BA because of infection screening, safety, and infant–host vulnerability concerns
Butyrate/SCFA–directed therapy	Preclinical or exploratory; timing and dose vary across models [27,107,108]	Experimental reduction in bile duct inflammation–, fibrosis–, and dysbiosis–related injury	Mechanistically attractive, but no standardized pediatric dose or hard clinical outcome data are available
UDCA	Successful post–KPE cohort treated for at least 18 months [109]; historical cohort often used 20 mg/kg/day for mean 252.6 days [110]	Improved postoperative liver biochemistry in selected children [109]; conflicting cohort–level outcome data [110]	Stage and patient selection appear critical; current evidence does not establish improvement in native liver survival or transplantation–free survival
Other metabolites	EPA or D–2–hydroxyglutarate–related approaches remain exploratory [111,112]	Mainly biochemical or mechanistic signals	Potentially informative, but too early for clinical recommendation

Abbreviations: BA, biliary atresia; FMT, fecal microbiota transplantation; HPE, hepatoportoenterostomy; KPE, Kasai hepatoportoenterostomy; LGG, Lactobacillus casei rhamnosus/Lactobacillus rhamnosus GG, as named in the source studies; SCFA, short–chain fatty acid; and UDCA, ursodeoxycholic acid.

## 8. Current Limitations and Future Perspectives

Current evidence remains insufficient to define whether dysbiosis is a driver of BA onset, a consequence of cholestasis, or both. Most studies are small, single–center, observational, and heterogeneous with respect to age, feeding status, antibiotic exposure, geography, sequencing platform, and pre– versus post–KPE sampling. These limitations complicate causal inference and weaken cross–study comparability.

The next phase of the field should prioritize longitudinal, multicenter, stage–stratified studies that integrate metagenomics, metabolomics, and clinically relevant endpoints. Biomarker studies should report externally validated performance metrics, and intervention trials should specify dose, timing, duration, and primary endpoints up front. Without this level of rigor, microbiome findings will remain interesting but difficult to translate.

Effective therapy in BA will probably require more than pathogen suppression alone; it will also require restoration of barrier–supportive, immune–regulatory, and bile acid–balancing microbial functions. This mechanistic view may help guide more rational microbiota–based precision strategies in the future.

## 9. Conclusions

The gut microbiota in BA has both harmful and protective associations. Dysbiosis weakens the intestinal barrier, increases portal delivery of MAMPs, intensifies immune and oxidative injury, disrupts bile acid signaling, and accelerates fibrosis. In contrast, beneficial microbial communities and their metabolites preserve epithelial integrity, support immune tolerance, and help maintain metabolic homeostasis.

Available biomarker and therapeutic studies are promising but not yet sufficient for routine clinical use. The most important next step is not simply to catalog additional taxa, but to validate stage–specific microbial signatures and interventions against hard outcomes such as jaundice clearance, recurrent cholangitis, native liver survival, and transplantation–free survival.

## Data Availability

No new data were created or analyzed in this study.
